# Growth and Geographic Variation in Hospitalizations with Resistant Infections, United States, 2000–2005

**DOI:** 10.3201/eid1411.080337

**Published:** 2008-11

**Authors:** Marya D. Zilberberg, Andrew F. Shorr, Marin H. Kollef

**Affiliations:** Evi*Med* Research Group, LLC, Goshen, Massachussetts, USA (M.D. Zilberberg); University of Massachusetts, Amherst, Massachusetts, USA (M.D. Zilberberg); Washington Hospital Center, Washington, DC, USA (A.F. Shorr); Barnes Jewish Hospital, St. Louis, Missouri, USA (M.H. Kollef)

**Keywords:** MRSA, CDAD, VRE, Pseudomonas, Candida, antibiotic resistance, epidemiology, hospitalizations, regional variations, dispatch

## Abstract

From 2000 through 2005, hospitalizations with resistant infections (methicillin-resistant *Staphylococcus aureus*, *Clostridium difficile–*associated disease, vancomycin-resistant enterococcus, *Pseudomonas aeruginosa,* and *Candida* infection) nearly doubled, from 499,702 to 947,393. Regional variations noted in the aggregate and by individual infection may help clarify modifiable risk factors driving these infections.

Over the past decade we have witnessed a rise in the antimicrobial drug–resistance epidemic in the United States and worldwide. Not only are resistant organisms such as methicillin-resistant *Staphylococcus aureus* (MRSA) and *Clostridium difficile* encountered with increasing frequency (*1*,*2*), but as their susceptibility to antimicrobial agents is waning, their overall virulence is on the rise (*3*,*4*). Additionally, though traditionally thought of as a nosocomial pathogen, MRSA, for example, is now a well-recognized community-acquired infection (*5*).

With the rapid growth of resistance, and the added associated illnesses and deaths (*6*,*7*), these infections exert a considerable strain on the US healthcare system, specifically on hospitals. Although important to understand for individual infections, the aggregate volume of resistance is an important factor in illustrating the problem as a whole and in helping identify the potential resources needed to deal with the epidemic. To understand the full extent of resistant infections in US hospitals, we examined their longitudinal trends from 2000 through 2005, focusing further on regional patterns of resistance during this time frame.

## The Study

We identified all hospitalizations carrying a diagnosis of MRSA, *Clostridium difficile*–associated disease (CDAD), vancomycin-resistant enterococcus (VRE), *Pseudomonas aeruginosa,* and *Candida* infections for 2000–2005 from the National Inpatient Sample data. These data are available on the Healthcare Costs and Utilization Project net [HCUPnet] website, administered by the Agency for Healthcare Research and Quality (*8*). We used the corresponding diagnosis codes from the International Classification of Diseases, 9th revision, Clinical Modification (Appendix Table). Because few reports of vancomycin-resistant *Staphylococcus aureus* exist (*9*), we assumed that most cases with the code V09.8 represented VRE infections. We limited hospitalizations in which *Candida* organisms had been identified to deep-seated infections, including candidiasis of the lung, disseminated candidiasis, candidal endocarditis, meningitis, esophagitis, and enteritis. The numbers of discharges per year for infections associated with each organism and in aggregate were stratified by census region. We obtained regional estimates of all US hospitalizations in the corresponding years from the HCUPNet (*8*), and censal and intercensal data on the US population for 2000–2005 from the US Census Bureau. We calculated region-specific hospitalization incidence rates associated with the resistant pathogens. Because large numbers would predispose the study to type I error, we did not perform formal significance testing; rather, we focused on clinical and policy-relevant trends.

The overall volume of resistant infections increased by 89.6% from year 2000 through 2005 (Table). As a proportion of the total volume growth, the increases across regions were comparable. The southern region had the highest raw volume of resistant infections for the study period (2000, 37.3%; 2005, 39.1%). The West had the smallest contribution in 2000 (19.0%) and 2005 (19.5%). However, the Northeast had the highest relative incidence per 1,000 hospitalizations with 14.00 in year 2000; its incidence of 19.98 in 2005, however, was lower than that in the South, 20.76/1,000 (Table). Regional disparities in the population-based incidence of hospitalizations with resistant organisms also occurred (Table). Thus, the incidence in the Northeast was not only the highest for 5 of the 6 years examined, but compared to that seen in the lowest-incidence region, the West, was higher by as much as 41.9% in 2003. This gap shrank in 2004 and 2005 to 29.9% and 27.7%, respectively.

**Table:** Volume, incidence of, and hospitalizations for infections with resistant organisms in the United States, by census region, 2000–2005

Hospitalizations and incidence	2000	2001	2002	2003	2004	2005
Annual no. hospitalizations						
All US	499,702	559,728	639,468	699,140	783,601	947,393
Northeast	102,913	119,799	132,607	151,306	152,881	188,306
Midwest	115,623	122,122	144,647	161,166	179,547	204,351
South	186,320	212,450	245,933	256,420	305,822	370,348
West	94,846	105,357	116,281	130,247	145,353	184,390
Incidence/1,000 hospitalizations						
All US	13.72	15.05	16.92	18.29	20.27	24.19
Northeast	14.00	16.17	17.74	20.01	19.98	24.29
Midwest	13.72	14.10	16.47	18.27	20.14	22.65
South	13.59	15.04	17.09	17.62	20.76	24.79
West	13.69	15.07	16.25	17.88	19.74	24.76
Incidence/100,000 population						
All US	177.08	196.32	222.12	240.71	267.27	320.18
Northeast	191.76	222.22	244.98	278.51	280.73	345.49
Midwest	179.27	188.40	222.21	246.63	273.59	310.31
South	185.27	208.55	238.33	245.54	288.87	344.88
West	149.45	163.31	177.59	196.33	216.17	270.56

When the incidences of individual component infections were examined, several patterns emerged. While the Northeast led other regions in the incidence of CDAD hospitalizations over the entire period examined (Figure, panel A), the South exhibited the highest population incidence of MRSA and *Pseudomonas* hospitalizations. Although temporal patterns of regional population incidence varied somewhat for hospitalizations in which VRE and *Candida* spp. infections were diagnosed, by year 2005 the Northeast emerged as the region with the highest incidence of VRE, while the South had the highest incidence of *Candida* spp. hospitalizations. The lowest incidence of VRE hospitalizations was consistently seen in the southern region in each of the studied years. The incidence of hospitalizations with pseudomonal infections remained relatively stable regionally over time (Figure, panels **B**, **C**, **D**).

**Figure:**
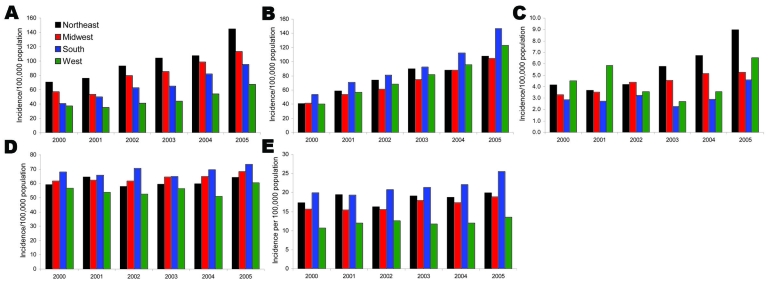
Population incidence of component resistant infections in the United States, by census region, 2000–2005. A) *Clostridium difficile–*associated disease; B) methicillin-resistant *Staphylococcus aureus*; C) vancomycin-resistant enterococcus; D) *Pseudomonas aeruginosa*; E) *Candida* spp.

## Conclusions

We have demonstrated a substantial rise in the absolute number, incidence, and geographic variations across the United States in hospitalizations in which infections have been caused by pathogens exhibiting antimicrobial resistance. The Northeast consistently outpaced the other regions in the aggregate volume of resistant infections in 5 of the 6 years examined. For individual infections, a region’s having a relatively high incidence of 1 organism does not guarantee it will have a high incidence of another organism, as illustrated by the reversal of the regional incidence patterns for MRSA and CDAD, for example. Most troubling, however, is the general finding of a ubiquitous, substantial, and continuing increase in the incidence of hospitalizations with resistant infections.

A notable pattern in our study is that the regions with the higher incidence of CDAD (Northeast and Midwest) also exhibited higher incidence of VRE in at least half of the study period, consistent with the observation that infection with CDAD can facilitate transmission of VRE (*10*,*11*). The South had the highest incidence of MRSA and lowest incidence of VRE. Since both pathogens share similar risk factors, why this pattern should be present is biologically unclear (*12*,*13*), although a recent report noted a similar pattern of concomitant increases in MRSA and decreases in VRE incidence between 1999 through 2005 (*14*). This potential inverse relationship should be investigated further. Lastly, we noted that, although substantially discrepant regionally, the incidence of hospitalizations with *P. aeruginosa* infections, consistent with others’ observations, has remained relatively stable over the 6-year period (*15*). We cannot illuminate the reasons for the patterns of infection incidences we have uncovered. Further studies should encompass much more granular geographic data to confirm our findings and raise hypotheses to explain them.

The most important limitation of our study is that case ascertainment was performed by using administrative coding, rather than clinical and microbiologic data, and we were unable to verify diagnostic accuracy either across time or geographic areas; therefore, the observed increases may be partially due to increased awareness of resistance. However, administrative coding has been used to track the epidemiology of both MRSA and CDAD (*1*,*2*). Furthermore, temporal trends in case volume are similar to trends reported from clinical studies. At least a proportion of the case-patients we identified likely had overlapping infections with multiple organisms. Nevertheless, the aggregate number of infections that we have described has implications for hospital resource use because persons with multiple infections likely require more care than those with a single pathogen. Finally, we were unable to differentiate between community-acquired and nosocomial infections.

In summary, we have demonstrated a notable increase in the incidence of hospitalizations with resistant organisms in the United States. Regional variations in the incidence may yield clues for future research efforts to ascertain what modifiable risk factors drive decreases in the incidence of these deadly infections. The nearly 1 million annual hospitalizations in 2005 with resistant infections and their relentless upward trajectory in the United States are undesirable and unsustainable. Aggressive and coordinated efforts to reduce inappropriate use of antibimicrobial agents in humans and livestock and to encourage development of novel therapeutics are urgently needed to stem this public health hazard in the United States and throughout the world.

## Supplementary Material

Appendix Table ICD-9-CM codes*
