# Supplementary material for: Growth and Geographic Variation in Hospitalizations with Resistant Infections, United States, 2000–2005
Source: Emerg Infect Dis. 2008 Nov;14(11):1756–8. doi: 10.3201/eid1411.080337 (PMC2630735; doi:10.3201/eid1411.080337)
Supplement: Appendix Table — ICD-9-CM codes* [file 08-0337_appT-s1.pdf]

Appendix Table. ICD-9-CM codes*

Organism or disease	ICD-9-CM code	Explanation
MRSA	V09.0	Infection with a microorganism resistant to penicillin
CDAD	8.45	Intestinal infection with *Clostridium difficile*
VRE	V09.8	Infection with microorganisms resistant to other specified drugs, including 1) vancomycin (glycopeptide) intermediate *Staphylococcus aureus* , 2) vancomycin (glycopeptide)-resistant enterococcus, and 3) vancomycin (glycopeptide)-resistant *Staphylococcus aureus*
Pseudomonas aeruginosa	008.42	*Pseudomonas* enteritis
	038.43	*Pseudomonas* septicemia
	041.7	*Pseudomonas* infection, not otherwise specified
	482.1	Pseudomonal pneumonia
Candida spp.	112.4	Candidiasis of the lung
	112.5	Disseminated candidiasis
	112.81	Candidal endocarditis
	112.83	Candidal meningitis
	112.84	Candidal esophagitis
	112.85	Candidal enteritis

*ICD-9-CM, International Classification of Diseases, 9th revision, Clinical Modification; MRSA, methicillin-resistant *Staphylococcus aureus*; CDAD, *Clostridium difficile*–associated disease; VRE, vancomycin-resistant enterococcus.
